# Lack of communication between healthcare professionals and women with ovarian cancer about sexual issues

**DOI:** 10.1038/sj.bjc.6600799

**Published:** 2003-03-04

**Authors:** M L Stead, J M Brown, L Fallowfield, P Selby

**Affiliations:** 1 National Cancer Research Network Co-ordinating Centre, Arthington House, Hospital Lane, Leeds LS16 6QB, UK; 2 Northern and Yorkshire Clinical Trials and Research Unit, 17 Springfield Mount, Leeds LS2 9NG, UK; 3 Sussex Psychosocial Oncology Group, Cancer Research UK, Brighton & Sussex Medical School, University of Sussex, Falmer BN1 9QG, UK; 4 Cancer Research UK Cancer Medicine Research Unit, St James's University Hospital, Beckett Street, Leeds LS9 7TF, UK

**Keywords:** ovarian cancer, sex behaviour, communication

## Abstract

Gynaecological cancer has been shown to affect women's sexual functioning, yet evidence suggests that healthcare professionals rarely discuss sexual issues with women diagnosed with a gynaecological cancer. Few studies have investigated why there is a lack of communication between healthcare professionals and women about sexual issues. Our study investigated the attitudes and behaviours of the 27 doctors and 16 nurses treating women with ovarian cancer in our centre towards the discussion of sexual issues, and also investigated women's experiences of such communication. Our findings showed that although most healthcare professionals thought that the majority of women with ovarian cancer would experience a sexual problem, only a quarter of doctors and a fifth of nurses actually discussed sexual issues with the women. Reasons for not discussing sexual issues included ‘it is not my responsibility’, ‘embarrassment’, ‘lack of knowledge and experience’ and ‘lack of resources to provide support if needed’. While some of these reasons were also viewed as barriers by the women, the results demonstrate that there is a need from the women's perspective to improve communication about sexual issues, although the most appropriate approach to this remains to be investigated.

Over 25 years ago, the *British Medical Journal* published two articles discussing sexual problems following gynaecological procedures (Amias, 1975a,1975b), stating that if women were encouraged to discuss such problems, then future stress could be avoided or reduced. However, despite evidence that women with gynaecological cancer experience alterations in sexual functioning (Andersen and Jochimsen, 1985; Andersen *et al*, 1989; Thranov and Klee, 1994; Bergmark *et al*, 1999), more recent publications demonstrate that communication between healthcare professionals and patients is still lacking (Corney *et al*, 1993; Cull *et al*, 1993; Lamb and Sheldon, 1994).

Few studies have investigated why healthcare professionals do not discuss sexual matters in gynaecological cancer. Studies show that oncology nurses rarely initiate discussions with patients about sexual functioning (Wilson and Williams, 1988; Cort, 1998), usually because of a lack of knowledge and communication skills. Many of the nurses believed that it was their responsibility to advise or help patients experiencing sexual difficulties, but few felt confident doing so. It has been suggested that when taking a sexual history in any patient, less sensitive issues such as reproductive and menstrual history should first be discussed followed by the more sensitive topic of sexual activity, to reduce the abruptness of the conversation (Lamb, 1990). This can provide a useful approach for initiating discussions about sexual issues. Sexual issues do not have to be raised as a separate topic and can also be included in the routine discussions with women.

In a recently reported study of specialist nurses attending a communication skills training programme, 18% of the 129 nurses cited discussing sexual matters as their most challenging communication problem (Fallowfield *et al*, 2001), highlighting a need for further education. Self-confidence in discussing sex increased significantly after a 2-day training course (*P*<0.0001). A recent study (Maughan and Clarke, 2001) piloted the use of a clinical nurse specialist in providing pre- and postoperative psychosexual counselling, and found that sexual functioning was improved compared with no counselling, demonstrating the benefits of such interventions.

It is suspected that doctors also rarely initiate discussions with patients about sexual issues, because of lack of time, embarrassment and lack of communication skills in this sensitive and personal area (Corney *et al*, 1993; Cull *et al*, 1993). The reluctance of patients to ask about sexual activity may lead healthcare professionals to believe that a patient has adapted to any changes in sexual functioning, or that they have no sexual concerns (Barni and Mondin, 1997). It is also possible that healthcare professionals avoid discussing sexual issues to prevent uncovering issues with which they feel unable to handle.

It has been suggested that patients' misunderstandings, misconceptions and lack of information not only confound the problem, but also play a part in causing it (Burke, 1996), highlighting a need to provide good information. The provision of information may improve patients' satisfaction with their care and adjustment to the diagnosis (Thomas *et al*, 1999). Recent guidelines, *Improving Outcomes in Gynaecological Cancer*, the manual (NHS Executive, 1999), stress the importance of providing general information and also support to women about psycho-sexual issues.

Treatments for gynaecological cancer vary in severity and modality. Radiotherapy is often given to women with cervical cancer, but rarely in ovarian cancer. Surgery for ovarian cancer does not involve the removal of external genitalia, unlike surgery for vulval cancer. The different gynaecological cancers affect different age groups, with ovarian cancer generally affecting older women than cervical cancer. For these reasons, we chose to concentrate on ovarian cancer only, and investigated the attitudes and behaviours of the doctors and nurses treating women with ovarian cancer in our centre towards the discussion of sexual issues. The aim was to identify the level of information currently provided, the opinions of the healthcare professionals about sexual issues, and any training needs of medical staff. The attitudes and experiences of women about the provision of information about sexual issues were also investigated. This research was recently reported in the *British Medical Journal* (Stead *et al*, 2001).

## MATERIALS AND METHODS

### Interviews with healthcare professionals

All doctors and nurses involved in the treatment and care of women with ovarian cancer were identified and asked for written consent to take part in the study. A semistructured interview schedule was developed to investigate the following issues:

- knowledge of the frequency and types of sexual problems/concerns in ovarian cancer,
- attitudes and behaviours towards discussing sexual issues and
- knowledge of written information currently available for women about sexual issues.

The answers to each question were summarised using simple frequencies. Qualitative comments were summarised.

### Interviews with women

Semistructured interviews were carried out with the 15 women who consented to participate in a parallel study of the impact of ovarian cancer on sexual functioning. These women were identified from a sampling survey of all women with ovarian cancer in our centre (*n*=72), who were invited to participate in the study if they were either sexually active or inactive for reasons potentially related to the cancer (*n*=23). The interview determined whether the woman had received verbal or written information about sexual issues, and if so by whom, when, what information was provided and whether the partner was involved. If no information was given, it was asked whether information should have been given and if so by who, when, what information, who should initiate and whether their partner should have been involved.

Local ethics committee approval was granted for the study. Data were analysed using the SPSS statistical package. Fisher's exact test was used to assess the association between the gender of the healthcare professional and communication with women. Tests were considered to be statistically significant at the 5% level of significance.

### Availability of written information

Cancer help organisations were contacted to determine the availability of written information about sexual issues for women with ovarian cancer.

## RESULTS

### Interviews with healthcare professionals

#### Subjects

All identified doctors and nurses agreed to be interviewed. Sixteen doctors (11 males and five females, median age 38 years, range=27–62) and 27 nurses (two males and 25 females, median age 34 years, range=22–52) from St James's University Hospital, the Leeds General Infirmary or Cookridge Hospital in Leeds were interviewed between July and October 1998. Five doctors (two surgeons, one specialist registrar and two senior house officers) worked in general gynaecology, five doctors (two surgeons and three senior registrars) worked in gynaecological oncology, five doctors (three consultants and two specialist registrars) worked in medical oncology and one consultant worked in clinical oncology. Five nurses worked in general gynaecology (three on a ward and two in outpatients), six worked in gynaecological oncology (all on a ward) and 14 worked in medical oncology (six staff nurses/sisters, four chemotherapy nurses and four outpatient nurses). One nurse was a research nurse working in medical oncology and one was a Macmillan nurse.

#### Sexual problems

Thirty healthcare professionals (70%) thought that at least 75% of women would experience sexual problems, six (14%) thought that 50–75% would experience problems, three (7%) thought that less than 50% would experience problems and four (9%) were not sure whether or not women would experience sexual problems. Thirteen healthcare professionals (30%) based this answer on no evidence.

Although a wide range of problems was given overall (see Table 1

**Table 1:** Types of sexual problem mentioned by healthcare professionals

**Sexual problem**	**Number of healthcare professionals who mentioned problem (maximum=43)**	**Number of doctors who mentioned problem (maximum=16)**	**Number of nurses who mentioned problem (maximum=27)**
Decreased sexual desire	25	13	12
Fear	21	6	15
Dyspareunia	18	7	11
Partner problems	17	2	15
Decreased self-esteem/altered body image	15	6	9
Altered arousal/vaginal dryness	13	2	11
Tiredness	7	1	6
Fertility	3	0	3
Altered pleasure	2	1	1
Altered frequency of sexual activity	1	0	1
Menopausal symptoms	1	0	1
Malaise	1	1	0
Anger if partner wants sex	1	1	0
Not sure	1	1	0

), the majority of healthcare professionals were only aware of four or less different problems, and some problems were only mentioned by one or two healthcare professionals.

#### Attitudes of all healthcare professionals about the need for communication about sexual issues

Forty-two healthcare professionals thought that sexual issues should be discussed. The clinician who thought that sexual issues should not be discussed believed that healthcare professionals should wait until patients asked about sexual activity. Five doctors and six nurses thought that they personally should talk to patients; the others thought it was someone else's responsibility. No pattern emerged as to whose responsibility this was. Ten healthcare professionals thought that speciality was irrelevant: knowledge and good communication skills were more important.

Most healthcare professionals (41 out of 43) felt that patients should: be informed that sexual problems may arise, be reassured that sexual activity would not cause damage or a recurrence, be told that changes in sexual functioning were normal, be given advice about different sexual positions or the use of lubricants and be told that they would be offered help or counselling if a problem arose. Some healthcare professionals felt it would be useful to have research evidence to assist such discussions. It was also felt that partners needed information and advice.

#### Demographics of healthcare professionals discussing/not discussing sexual issues

Nine healthcare professionals (four out of 16 (25%) doctors and five out of 27 (19%) nurses) discussed sexual issues (see Table 2

**Table 2:** Demographics of healthcare professionals with respect to whether or not they discussed sexual issues with patients

**Characteristic**	**Discussed sexual issues**	**Did not discuss sexual issues**
*Age in years (median, range)*		
Doctors (*n*=16)	41 (29–56)	38 (27–62)
Nurses (*n*=27)	32 (26–46)	34 (22–52)
		
*Time since qualification in years (median, range)*		
Doctors (*n*=16)	14.5 (6–32)	14 (4–39)
Nurses (*n*=27)	8 (5–22)	12.5 (1–31)
		
*Gender of clinician/nurse (number, per cent)*		
Male doctors (*n*=11)	3 (27%)	8 (73%)
Female doctors (*n*=5)	1 (20%)	4 (80%)
Male nurses (*n*=2)	1 (50%)	1 (50%)
Female nurses (*n*=25)	4 (16%)	21 (84%)
All male healthcare professionals (*n*=13)	4 (31%)	9 (69%)
All female healthcare professionals (*n*=30)	5 (17%)	25 (83%)

for demographics). Two doctors who discuss sexual issues worked in gynaecological oncology (one surgeon, one senior registrar), one worked in medical oncology as a specialist registrar and one was a consultant in clinical oncology. Two nurses who discuss sexual issues worked on a general gynaecology ward, two on a medical oncology ward and one was the Macmillan nurse. A similar proportion of staff working in gynaecology (four out of 21) compared with medical oncology (three out of 20) discussed sexual issues. Although it appeared that more males than females discussed sexual issues with women (31% compared to 17%), this was not statistically significant (*P*=0.42).

#### Behaviours of healthcare professionals

Although 98% of healthcare professionals thought that sexual issues should be discussed, only 21% discussed sexual matters. Of the nine who discussed sexual issues, only three talked to all women, with six discussing sexual issues with less than half of their patients (usually excluding elderly or very ill women). The frequency of discussions was often limited to once before surgery and once after surgery. Seven healthcare professionals asked a general question about sexual problems and told women when it was safe to resume sexual activity. The other two discussed specific issues, such as fertility, but only if they thought it was necessary. Partners were involved on an *ad hoc* basis.

Healthcare professionals gave the following responses about talking about sexual issues:

- It is easier with younger patients.
- The subject is easy to avoid, but should be addressed.
- You need to feel confident talking to patients.
- It is part of the job, so I expect to talk to patients.
- I feel comfortable about it, sex is a bodily function like all others that are discussed.

The most frequent reasons for not discussing sexual issues were ‘it is not my responsibility’ or ‘it is not appropriate for me to talk to patients'. Nurses often only saw women during chemotherapy or on the ward after surgery, or they did not see women alone. Two doctors felt that sexual problems were not entirely medical and that it was the role of nurses to discuss counselling issues. One clinician felt that sexual functioning was not perceived to be a significant clinical problem and that doctors were usually pleased to see women alive in clinic; sexual functioning was not relevant. Other reasons for not talking to women included:

- embarrassment,
- lack of privacy,
- limited time,
- should wait until the patient asked about sex,
- lack of knowledge/experience/skills,
- lack of resources to provide support if a problem was identified,
- most patients too old,
- low priority at diagnosis and during treatment,
- taboo subject and
- never thought about it before.

A number of additional comments were made when asked why there was no communication about sexual issues, including:

- There is no role model to follow.
- You would be opening a can of worms.
- Patients may be taken aback if they were asked about sex.
- People do not talk about those sort of things.
- It is medical tradition not to ask.

Half of the healthcare professionals who did not talk to women were not sure whether anybody else in their hospital did. One nurse from a medical oncology ward expected gynaecology ward staff to discuss sexual issues with women. There were mixed feelings about when sexual issues should be discussed.

#### Knowledge of written information for patients

Only two doctors (12%) and eight nurses (30%) were aware of any written information for patients about sexual matters.

### Interviews with women

#### Subjects

Fifteen women (median age 56 years, range=42–71; median time since diagnosis 18 months, range 8–120) were interviewed between November 1998 and February 1999. Twelve women had been sexually active prior to the diagnosis, 10 of whom (83%) experienced an alteration in their sexual functioning after the diagnosis (see Table 3

**Table 3:** Types of sexual problem experienced by women who were sexually active prior to the diagnosis

**Sexual problem**	**Number of women for whom problem is relevant**	**Number (%) of women experiencing problem**
Decreased frequency of sexual activity	12	9 (75%)
Decreased sexual desire	12	8 (67%)
Fear	12	5 (42%)
Decreased self-esteem/altered body image	12	5 (42%)
Tiredness	12	4 (33%)
Menopausal symptoms	12	1 (8%)
Fertility	12	1 (8%)
Altered pleasure	9	6 (67%)
Dyspareunia	9	4 (44%)
Altered arousal/vaginal dryness	9	3 (33%)

).

#### Experience of communication

No woman had received written information and only two received brief verbal information: a medical oncologist told one woman that if intercourse proved difficult the hospital had creams to help; another woman had a vague recollection of a surgeon saying something, but she still felt unsure about the safety of sexual activity. The women thought that written information would help prepare people, and that answers to questions guidelines on how sexual functioning was normally affected, a named contact for questions, or a telephone number of a helpline should be given.

#### Attitudes towards discussing sexual issues

Eleven women (73%) felt that verbal information should be provided:

- I thought it might hurt and unless someone talks to you and tells you these things you don't know.
- I didn't know much about how sex would be affected, I just had to go through and find out for myself.
- You should know what's going to happen instead of it hitting you like a ton of bricks.
- You have no idea about how the cancer will affect you sexually.
- Nobody talks about sex and you wonder whether it is right that you feel different.
- I wondered how other people were affected.
- You should be able to discuss sex if you have problems or if they think you will have a problem.
- You should be offered information and then decide whether to take it up. I would have wanted it if it was offered.

Nine women who felt that sexual issues should be discussed thought that the hospital staff should initiate the discussion. One woman said, ‘Until you get home you do not wonder whether you will have any sexual problems, so how can the patient be expected to ask?’

Five women said that it would not matter whether a man or women spoke to them about sexual activity, with a further six preferring a woman to talk to them. Nine women said the age of the person talking to them did not matter, although two would have preferred someone older to talk to them about sexual issues. There were varying opinions of who should discuss sexual issues, when the discussions should occur and whether or not partners should be involved.

#### What should be discussed

The women would have liked to have been told whether or not changes in sexual functioning were normal and expected, to have been given examples of the types of problem which may occur and when, to have been reassured that intercourse was safe and to have been given the opportunity to ask questions. It seemed that the women were not seeking long discussions about sexual functioning – many felt that a few minutes discussion or a mention that sexual functioning could be affected was all that was needed. It was suggested that the effects of treatment on sexual functioning could be included with the list of more frequently discussed side effects such as nausea and vomiting.

#### Benefits of discussing sexual issues

Potential benefits of communication included:

- *Understanding that problems were normal* – ‘it would make you think that because other women have gone through it, what you're going through is nothing different, otherwise you feel that you are different. It gives you confidence if you know others are experiencing the same problems'.
- *Knowledge of the cause and duration of problems* – ‘I could have understood why I was having sexual problems if they'd have said “you might have problems sexually because we've removed this or that’.
- *Provides an opportunity and permission for women to ask questions about sexual activity* – ‘if you are told before treatment starts that sex will be affected, you can think about questions to ask when treatment has finished’.
- *Helps to reduce anxiety caused by problems* – ‘if sex is discussed at the beginning, it's one less thing to be a burden along with everything else’.
- *Helps to improve communication with partners* – ‘you could discuss with your partner what would happen’.

### Availability of written information

CancerBACUP produce two booklets: *Sexuality and Cancer* and *Understanding Cancer of the Ovary*. The first describes male and female anatomy and discusses the different phases of the sexual response cycle and how these may be affected by the cancer and its treatment. The second is ovarian cancer specific, and includes a section about the sexual effects of surgery. Although brief, it discusses when it is safe to resume a sexual relationship after a hysterectomy, fertility issues, physiological difficulties such as vaginal dryness and decreased sexual desire.

CancerLink produce a booklet entitled *Body Image Sexuality and Cancer*, which discusses barriers to communication about sexual relationships, body image, how cancer can affect feelings and relationships, and how treatment may cause sexual problems.

Ovacome is an ovarian cancer support group that produces regular newsletters to provide a forum for women to share personal experiences and provide information about the disease and its treatments. There were no articles about sexual activity in the newsletters prior to the presentation of the results of this study in the winter 2002 edition, although fertility had been discussed in a previous newsletter. Since this study was carried out, Ovacome have also produced a factsheet entitled *Ovarian Cancer and Your Sexuality*.

## DISCUSSION

Although the sample size for this study was relatively small, our centre is large and has a mixed community. The findings are so extreme that some conclusions appear to be very likely, despite the limitations of the localised sample. This is the first study investigating the attitudes and behaviours of a wide group of healthcare professionals towards communication about sexual functioning, and also the first to investigate the information needs of women with ovarian cancer. Most healthcare professionals thought that the majority of women would experience sexual problems, although this was usually an educated guess based on no evidence. However, only a quarter of doctors and a fifth of nurses did discuss sexual issues. Except on the rare occasions when a woman had initiated a discussion about sex, the remaining healthcare professionals never discussed sexual functioning with women. This seemed to correlate with the women's experiences. The clinician's age and the time since qualification did not appear to affect communication about sexual issues. Similarly, age did not seem to influence whether or not nurses discussed sexual issues. However, it appeared that newly qualified nurses and nurses who had been in post for a longer time did not discuss sexual activity.

The age and health status of the patient were raised as barriers to discussing sexual issues. However, evidence from our study and previous research (Starr and Weiner, 1981; Shell and Smith, 1994) suggests that it is not unusual for women over the age of 60 years to continue with a sexual relationship. The assumption that women will be sexually inactive during chemotherapy was not supported in our study. Having sexual activity during treatment helped one woman cope with the treatment and its side effects and contributed to feelings of self-esteem, femininity and confidence. While sensitivity may be needed when approaching the subject of sexual activity in women undergoing treatment, it is not acceptable to assume that sexual functioning is not important during this time.

Other reasons for not discussing sexual issues included a lack of knowledge and skills, and embarrassment, highlighting the need for sexual education. Reasons such as lack of time, lack of privacy and lack of resources highlight the need for more practical changes, which unfortunately may be more difficult to implement. Women would like an identified person to contact should sexual problems occur or should there be concerns about sexual functioning. The cost-effectiveness of such an intervention would require proper evaluation.

The behaviour of most healthcare professionals did not correlate with their attitude towards discussing sexual issues. Healthcare professionals who did not discuss sexual issues with women thought that someone should, although most thought this was the responsibility of another member of staff. Although there was not a tendency for doctors to expect nurses to talk to women, and *vice versa*, there was evidence that healthcare professionals working in general oncology settings expected gynaecology staff to be more familiar with discussing such issues. However, our findings did not confirm this expectation. Our study highlighted a need for improved communication between disciplines within individual hospitals. Integrated care and communication across multidisciplinary teams to improve patient care are requirements of the Calman-Hine (1995) report and the more recent NHS Cancer Plan (Department and Health, 2000); however, the findings of this study suggest that these recommendations are not yet being met. Most healthcare professionals were unaware of whether or not other colleagues discussed sexual issues with women. It is recommended that healthcare professionals within individual hospitals develop strategies for communication about sexual issues, including who will talk to women about sexual issues and when.

Perceived benefits of discussing sexual issues included adequate preparation for alterations in sexual functioning, reassurance that problems were normal, reassurance that sexual activity would not cause a recurrence and legitimising sexual concerns and questions. More practical help or advice, such as the provision of lubricants or suggesting alternative sexual positions may help reduce any pain or discomfort, which may enable women to return to a better sex life quicker.

There was little knowledge of written information about sexual issues for patients with cancer. No patient had received any written information, although most thought it would be useful. This is despite the fact that booklets about sexuality are produced by CancerBACUP, CancerLink and Ovacome, specifically for patients with cancer. The use of these booklets is recommended.

The potential limitations of this study warrant consideration. The research was based only in one city, and therefore may not reflect attitudes and opinions of healthcare professionals across the UK. However, Leeds is a large centre and the study involved *all* clinicians and nurses treating patients with ovarian cancer in the three major cancer hospitals in Leeds, thus all disciplines were represented, and it is likely that their views and patterns of communication will be similar to those of healthcare professionals in other UK cities. Leeds has a mixed community and so the women sampled were probably typical of the ovarian cancer population across the UK. However, the study possibly involved a somewhat selected group of women – those who were willing to complete the survey questionnaire and, of those, women who were either sexually active or inactive for reasons potentially related to the cancer. Again though, it is likely that the women included in the study were representative of women with ovarian cancer in terms of the level of communication with healthcare professionals about sexual issues, as the healthcare professionals themselves also reported low levels of communication about sexual matters.

The information provided by the women showed the extent to which sexual functioning could be affected by a diagnosis of ovarian cancer and its treatment. The impact of these changes on the woman's and partner's lives at a time when their lives had already been turned upside down was unsatisfactory, as was the fact that healthcare professionals appear to be providing little or no support to women regarding sexual issues. It is evident from this study that sexual functioning remains a neglected aspect of oncology care. The reluctance of women to discuss concerns about sexual functioning with anyone, in particular their partner, appeared to have a major impact on the outcome on sexual activity, highlighting the need for improved support for these women.

The results from this study and the associated, parallel sexual functioning study demonstrate that sexual functioning is an area of QOL which undergoes disruption following a diagnosis of ovarian cancer, but one about which little is discussed. Sexual functioning is an important aspect of the QOL and well-being of many people, and any adverse change in sexual functioning may result in emotional disturbance and marital disharmony. At a time when women are having to adapt to life as a cancer patient, which brings its own emotional, physical and psychological disturbances, the experience of concerns, misunderstandings or fears about sexual activity represents additional and, more importantly, potentially avoidable stressors. While some of the healthcare professionals' reasons for the lack of communication about sexual issues were also viewed as barriers by the women (such as embarrassment and lack of time), the results demonstrate that there is still a need from the women's perspective to improve communication about sexual issues, although the most appropriate approach remains to be investigated. Sexual activity may not be the main concern of a woman diagnosed with ovarian cancer. People *can* live without sex. However, it must be recognised that sexual activity is an important component of the QOL and well-being of many people's lives, and is one which deserves attention from medical staff.

We are now planning a programme of research to identify the prevalence of sexual problems following a diagnosis of gynaecological cancer. The research will also include the development and evaluation of an intervention programme for women experiencing sexual dysfunction.
